# MACROSCOPIC AND HISTOLOGICAL ANALYSIS ON ENDOSCOPICALLY RESECTED RECTAL LESIONS

**DOI:** 10.1590/0102-672020230015e1733

**Published:** 2023-06-30

**Authors:** Marcos Onofre Frugis, Carmen Australia Paredes Marcondes Ribas, Osvaldo Malafaia, Fernando Issamu Tabushi, Nicolau Gregori Czeczko

**Affiliations:** 1 Hospital 9 de Julho, Digestive Endoscopy Service – São Paulo (SP), Brazil; 2 Faculdade Evangélica Mackenzie do Paraná, Postgradute Program – Curitiba (PR), Brazil.

**Keywords:** Colorectal neoplasms, Adenoma, Endoscopic mucosal resection, Neoplasias colorretais, Adenoma, Ressecção endoscópica de mucosa

## Abstract

**BACKGROUND:**

Colorectal cancer is among the most common malignancies worldwide. Colonoscopy is the examination of choice for the prevention of CRC because of its great diagnostic and, especially, therapeutic capacity in relation to adenomatous lesions.

**AIMS:**

This study aimed to analyze the prevalence, macroscopic, and histological characteristics of polypoid rectal lesions resected through endoscopic techniques and assess whether endoscopic therapy is safe and efficient for treating lesions located in the rectum.

**METHODS:**

This is a retrospective observational study with an analysis of the medical records of all patients undergoing resection of rectal polyps.

**RESULTS:**

A total of 123 patients with rectal lesions were evaluated, with 59 men and 64 women of mean age 56 years. All patients underwent endoscopic resection: 70% with polypectomy and 30% with wide mucosectomy. Complete colonoscopy with removal of the entire rectal lesion occurred in 91%, while in 5% the preparation was inadequate and poor clinical conditions were an impeditive factor, and in 4% surgical treatment was indicated because there was an infiltrative lesion with central ulceration. Histological evaluation showed adenomas in 3.25%, hyperplasia in 7.32%, and hamartoma in 0.81%; low-grade dysplasia was identified in 34.96%, high-grade dysplasia in 51.22%, and adenocarcinoma in 1.63%, while one case (0.81%) was classified as erosion.

**CONCLUSIONS:**

Polyps in the rectum are common and were found in 37% of these colonoscopies. Adenomas with dysplasia were the most common form of Colorectal cancer . Therapeutic colonoscopy proved to be a safe and efficient method for the complete treatment of rectal lesions.

## RESUMO

**RACIONAL:**

O câncer colorretal (CCR) está entre as neoplasias mais comuns em todo o mundo. A colonoscopia é o exame de escolha para prevenção por sua grande capacidade diagnóstica e, principalmente, terapêutica em relação às lesões adenomatosas.

**OBJETIVOS:**

Analisar a prevalência, as características macroscópicas e histológicas das lesões polipoides retais ressecadas por técnicas endoscópicas e avaliar se a terapia endoscópica é segura e eficaz para o tratamento de lesões localizadas no reto.

**MÉTODOS:**

Estudo observacional retrospectivo com análise dos prontuários de todos os pacientes submetidos à ressecção de pólipos retais.

**RESULTADOS:**

Foram avaliados 123 pacientes com lesões retais: 59 homens e 64 mulheres com idade média de 56 anos. Todos os pacientes foram submetidos à ressecção endoscópica: 70% com polipectomia e 30% com mucosectomia ampla. A colonoscopia completa com retirada de toda a lesão retal ocorreu em 91%, enquanto em 5% o preparo foi inadequado e as más condições clínicas foram fator impeditivo, e em 4% o tratamento cirúrgico foi indicado por haver lesão infiltrativa com ulceração central. A avaliação histológica mostrou adenomas em 3,25%, hiperplasia em 7,32% e hamartoma em 0,81%; displasia de baixo grau foi identificada em 34,96%, displasia de alto grau em 51,22% e adenocarcinoma em 1,63%, enquanto um caso (0,81%) foi classificado como erosão.

**CONCLUSÕES:**

Pólipos no reto são comuns e foram encontrados em 37% das colonoscopias. Adenomas com displasia foram a forma mais comum de câncer colorretal. A colonoscopia terapêutica mostrou-se método seguro e eficiente para o tratamento completo das lesões retais.

## INTRODUCTION

Colorectal cancer (CRC) is the third leading cause of death in the United States. In Brazil, according to the annual report available on the website of the National Cancer Institute^15^, it is the second in incidence and the third in mortality, in both men and women. Its incidence has increased over the last 15 years worldwide. It is believed that diets rich in animal fat and increased consumption of processed and manufactured foods, together with low fiber intake and sedentary lifestyles, have contributed to this increase^3^.

Colonoscopy is certainly the most effective preventive method for directly combating preneoplastic lesions, that is, polyps of the colon and rectum^13^. The advent of image magnification and chromoscopy and the evolution of materials for endoscopic resections, along with improved training for endoscopists, have been contributing to prevention^2^.

Consequent to the implementation of the American Cancer Society Guidelines, which have made it routine to undergo preventive colonoscopy from the age of 50 years onward and among the direct relatives of patients with a history of CRC from the age of 40 years onward, it is believed that the incidence of this form of cancer should fall over the next few years^13,16^.

Rectal cancer presents characteristics that differ slightly from cancer of the rest of the colon, due to its more aggressive anatomopathological peculiarities, which are associated with higher morbidity and mortality for this type of lesion^6^.

Thus, the relevance of our current project comes from its analysis of the prevalence, size, and distribution of rectal lesions resected through endoscopy, with anatomopathological analysis, to ascertain the efficacy and safety of the treatment of the cases selected.

The aim of this study was to analyze the prevalence, size, age distribution, and anatomopathological distribution of rectal lesions resected through endoscopy and ascertain whether this method was safe and effective as a treatment for the cases selected.

## METHODS

This was a retrospective, observational, and cross-sectional study in which the aim was to assess rectal lesions that underwent endoscopic resection (polypectomy and mucosectomy), at the Endoscopy Service of Hospital 9 de Julho in São Paulo, Brazil. This study was approved by the Research Ethics Committee of the Mackenzie Presbyterian Institute and registered on the Brazil Platform (n° CAAE 23424719.8.0000.0103).

Out of a total of 3,790 colonoscopies, 123 cases of rectal lesions resected through endoscopy were selected in accordance with the inclusion and exclusion criteria of the current research project. In cases of suspected lesions, the routine preparation consisted of washing with physiological serum solution followed by chromoscopy using 0.4% indigo carmine, in order to study the surface of the lesion and more accurately delimit its margins^12^.

The inclusion criteria were that the rectal lesions should be greater than or equal to 10 mm in diameter, without endoscopic signs suggestive of submucosal infiltration, which were resected by means of endoscopy (polypectomy or mucosectomy). The exclusion criteria were situations of rectal lesions smaller than 10 mm in diameter, or lesions larger than 10 mm with endoscopic signs suggestive of submucosal infiltration or that did not rise after injection of saline solution into the submucosa to attempt mucosectomy. Such cases were referred for a more individualized investigation and appropriate treatment. Advanced tumors and other inflammatory lesions of the rectum were also excluded.

### Procedures

A Pentax EPKi endoscope with image magnification accessories and digital chromoscopy (i-Scan) was used. Indigo carmine was used to stain the lesions. Physiological solution and, if necessary, adrenaline 1-10000u were used for infiltration of the submucosa, in order to elevate the lesions and enable complete resection or resection in pieces (master pieces) using a conventional polypectomy loop. Te lesions were classified macroscopically in accordance with the Paris classification system^9^ and anatomopathologically in accordance with the Vienna classification system^5,14^.

### Statistical analysis

The data from the patients and their polyps were compiled in Excel and shared in the Epi Info software for analysis of the variables presented in the results.

## RESULTS

A total of 123 cases of rectal lesions greater than or equal to 10 mm in diameter were collected and analyzed. There were slightly more cases among women (52.03%) than among men (47.97%) over the period studied. However, this difference in proportions was only a numerical difference of five cases (i.e., men accounted for only five cases fewer).

Regarding age groups, the patients were categorized into six groups, as follows: group 1: 30–40 years; group 2: 41–50 years; group 3: 51–60 years; group 4: 61–70 years; group 5: 71–80 years; and group 6: 81 years or older.

Table 1 presents the analysis on the rectal lesions greater than or equal to 10 mm in diameter that were resected by means of endoscopy, according to age group.

**Table 1:** Distribution according to age group.

Age group	Frequency (%)
30–40 years	13 (10.57)
41–50 years	17 (13.82)
51–60 years	28 (22.76)
61–70 years	31 (25.20)
71–80 years	22 (17.89)
81 years	12 (9.76)
Total	123 (100)

Although the guidelines (clinical protocol) recommend that preventive action through colonoscopy should only start to be implemented above the age of 50 years, it should be noted that the present study revealed that 25% of the cases occurred at less than 50 years of age, which was a proportion similar to that of the group aged 61-70 years.

Regarding the data on lesion size that were collected, the patients were categorized into six groups as follows: group 1: 10 mm; group 2: 11–20 mm; group 3: 21–30 mm; group 4: 31–40 mm; group 5: 41–50 mm; and group 6: 51 mm (Table 2).

**Table 2:** Lesion size.

Size (mm)	n (%)	Size (mm)	n (%)
10 mm	46 (37.40)	31–40 mm	9 (7.32)
11–20 mm	47 (38.21)	41–50 mm	5 (4.07)
21–30 mm	11 (8.94)	51 mm	5 (4.07)
Total	104 (84.55)	Total	19 (15.45)
Final total	123 (100)		

**Figure 1:**
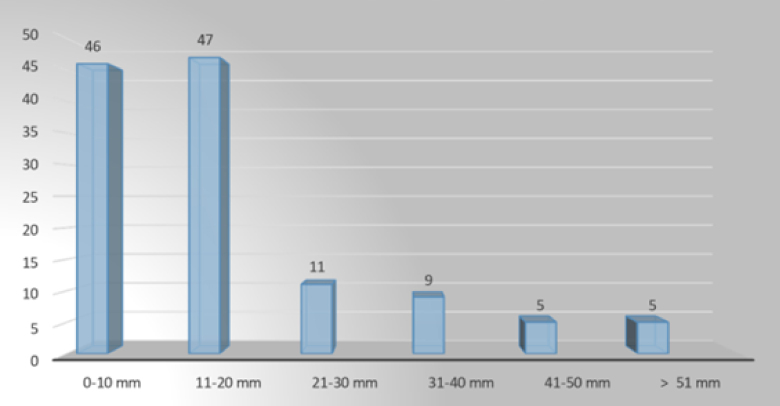
Lesion sizes.

Overall, regarding lesion sizes, lesions of sizes 10–30 mm were present in much higher quantities (84.55% of the total), and the highest proportion was the size range 11–20 mm (38.21%) (Figure 1).

The lesions were endoscopically classified using the Paris classification system^6^ (Table 3).

**Table 3:** Type of lesion according to the Paris classification system^6^.

Paris lesion classification	n (%)
IS	33 (26.83)
ISP	32 (26.02)
IP	8 (6.50)
IIA	9 (7.32)
IIA+IIC	2 (1.63)
LST	34 (27.64)
LST+IS	3 (2.44)
LST+ISP	1 (0.81)
LST+DEP	1 (0.81)
Total	123 (100)

IS: sessile; ISP: mixed; IP: pedunculated; IIA: nonpolypoid slightly elevated; IIC: nonpolypoid slightly depressed; LST: laterally spreading tumor; DEP: depressed.

We considered lesions of types 0-LST and 0-IIa (of the Paris classification^6^) together as superficially elevated lesions. Their surfaces were classified as granular when they presented mucosal granulation of up to 6 mm in diameter; nodular or mixed when one or more nodules of more than 6 mm in diameter were present; or smooth when the surfaces were smooth.

**Figure 2:**
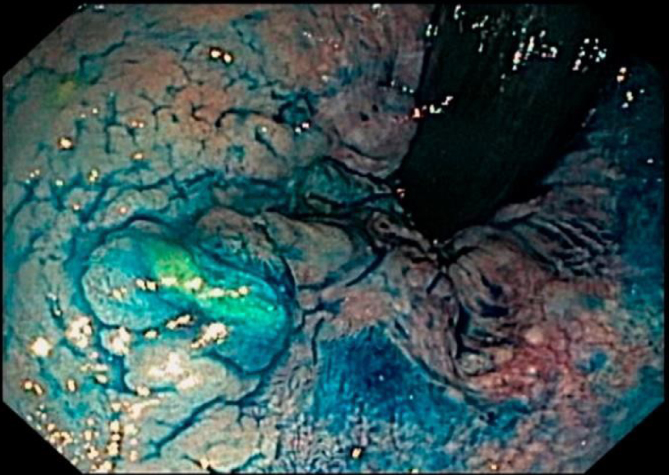
Rectal laterally spreading tumor (chromoscopy, Parada et al.^11^).

**Figure 3:**
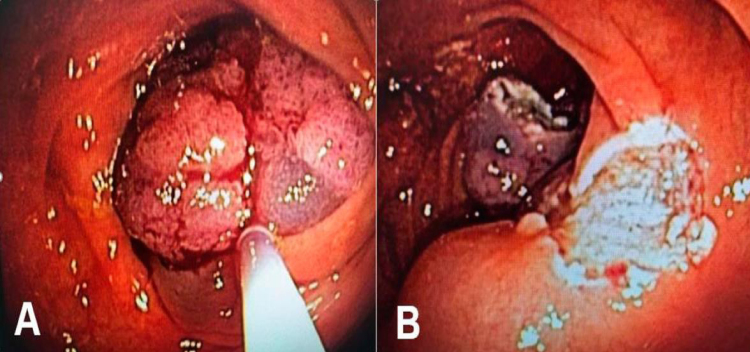
(A) Mucosectomy using rectal laterally spreading tumor loop. (B) Mucosa with margin.

With the evolution of concepts and with technological improvements, endoscopists have begun to diagnose more and more nonpolypoid or superficial lesions (superficially elevated, flat, or depressed) and lesions or tumors with spreading or lateral growth (laterally spreading tumors [LST]). These tend to grow laterally in relation to the surface of the colon or rectum, and by definition have a diameter greater than 10 mm^9,10^. In the present study, the prevalence of LST was 24.64%. These lesions invade the submucosa and therefore already present neoplastic cells; hence, they can no longer be treated endoscopically (Figures 2 and 3).

According to the Vienna classification system^5,14^ (Table 4), these lesions are histopathologically classified into the following five types:

**Table 4:** Anatomopathological categories according to the Vienna classification system^5,14^.

Anatomopathological categories	n (%)
Low-grade intraepithelial neoplasia (LG-IEN)	40 (32.52)
High-grade intraepithelial neoplasia (HG-IEN)	63 (51.22)
Low-grade sup intraepithelial neoplasia (LG-IEN-S)	3 (2.44)
Hyperplasia (HYPERPLA)	9 (7.32)
Carcinoma (CARCIN)	2 (1.63)
Erosion (EROSION)	1 (0.81)
Hamartoma (HAMART)	1 (0.81)
Adenoma SM1 (ADENSM1)	3 (2.44)
Adenoma SM2 (ADENSM2)	1 (0.81)
Total	123 (100)

SM: submucosal.

1. negative for dysplasia/neoplasia (includes reactive lesions);
2. undefined regarding dysplasia/neoplasia;
3. noninvasive low-grade intraepithelial neoplasia (LG-IEN), equivalent to low-grade dysplasia and corresponding to mild and moderate dysplasia in the three-grade system, that is, low-grade adenoma/dysplasia;
4. noninvasive high-grade intraepithelial neoplasia (HG-IEN), equivalent to high-grade dysplasia: adenoma with high-grade dysplasia or intense dysplasia in the three-grade system, noninvasive in situ carcinoma, and intramucosal carcinoma invading the lamina propria; and
5. invasive neoplasia, which invades as far as the submucosa or even more deeply^5,14^.

The HG-IEN was found in 51.22% of the cases, and four with adenocarcinoma were referred for complementary treatment (Figure 3).

From the point of view of treatment, patients whose lesions were limited to the mucosa can be considered to have been cured, strictly speaking. The risk of lymph node metastases increases with the depth of invasion of the submucosa^9,8^.

## DISCUSSION

Colonoscopy has been shown to be a very safe and effective method for diagnosing rectal lesions, as well as for implementing definitive treatment for the vast majority of lesions. It can be used for performing polypectomy and mucosectomy safely, with high technical quality^13^.

The present study demonstrated the aggressiveness of polypoid lesions of the rectum, with special attention to LST lesions, which are more likely to be more aggressive. LST lesions are typically larger than 10 mm in diameter.

Among the 123 patients analyzed, despite the fact that colonoscopy is a very safe and effective method, the characteristic that drew our attention most was their age. 25% of these patients studied were between 30 and 50 years of age and had been asymptomatic. Therefore, endoscopic evaluation for these patients would not be prescribed, according to current guidelines. However, it is still worth mentioning that 50% of this sample was 50–70 years of age. Regarding the size of the lesions, we took into consideration lesions that were at least 10 mm in diameter. This minimum diameter accounted for 37.4% of the sample. Lesions larger than 51 mm accounted for 4.07%. The mean size was 11–20 mm (38.21%). These findings are in agreement with those found in other similar studies, such as that of Heo et al.^7^, in which the mean size was 19.8±12.1 mm.

Intraepithelial neoplasia is a denomination introduced through the Vienna consensus of 2002 to replace the terms adenoma and dysplasia. It is subdivided into high and low grades. According to the WHO, in situ and intramucosal carcinoma should be classified as dysplasia or HG-IEN, because the repercussions of the lesions are exactly the same, that is, none of them have the potential to send metastases to other organs and lymph nodes^12^.

The precursor lesions include superficially elevated, flat, depressed, excavated, and lateral-growth lesions^4^. In the literature, it is stated that flat lesions have a higher chance of progression to HG-IEN, predominantly in the right colon and usually with a thickness of less than 1 cm. These present higher risk of infiltrative growth than sessile adenomas of the same size, especially in depressed lesions^1^.

In a study by Diger et al.^4^, out of the 200 patients evaluated, 21% of the colonic lesions and 56% of the rectal lesions were of high grade, compared with 51.22% of the rectal lesions that were of high grade in the present study. Among our patients who were identified as having HG-IEN, adenocarcinoma was found in 4 (3.25%). These patients were referred for further treatment. This number is slightly lower than that found in a study by Heo et al.^7^, in which 8.9% of the patients required additional treatment after endoscopic treatment.

In general, the results from studying these 123 patients were quite balanced in relation to the worldwide literature. Reiterating, there were no significant differences between the sexes, and the main point that needs to be highlighted is the issue that 25% of the cases were less than 50 years of age.

As demonstrated, among the 63 patients who benefited from endoscopic resection, 51.22% had HG-IEN, and 4 of them already had adenocarcinoma and were referred for complementary treatment. It is thus evident that colonoscopy is a method that should be increasingly used and, if possible, earlier.

## CONCLUSION

Colonoscopy, used in association with endoscopic mucosectomy, is a safe and very effective practice for diagnosing, treating, and following up rectal lesions.
